# The Global, Regional, and National Burden of Lower Respiratory Infections Caused by *Streptococcus pneumoniae* Between 1990 and 2021

**DOI:** 10.3390/healthcare13161982

**Published:** 2025-08-12

**Authors:** Zhenxuan Kong, Jin Xiong, Lin Chen, Kaicheng Peng, Hui Liu, Qinyuan Li, Zhengxiu Luo

**Affiliations:** 1 Department of Respiratory Children’s Hospital of Chongqing Medical University, National Clinical Research Center for Child Health and Disorders, Ministry of Education Key Laboratory of Child Development and Disorders, Chongqing 401122, China; 2022110405@stu.cqmu.edu.cn (Z.K.); xiongjin@stu.cqmu.edu.cn (J.X.); 2022140139@stu.cqmu.edu.cn (L.C.); 2022140140@stu.cqmu.edu.cn (K.P.); 2023130278@stu.cqmu.edu.cn (H.L.); liqinyuan@hospital.cqmu.edu.cn (Q.L.); 2 Key Laboratory of Children’s Important Organ Development and Diseases of Chongqing Municipal Health Commission, Chongqing 401122, China

**Keywords:** lower respiratory infection, *Streptococcus pneumoniae*, Global Burden of Disease, socio-demographic index, disability-adjusted life year

## Abstract

**Aims**: To investigate the global epidemiological characteristics of lower respiratory infection (LRI) burden caused by *Streptococcus pneumoniae* (SP) from 1990 to 2021. **Methods**: Using data from the Global Burden of Disease (GBD) study 2021, we systematically analyzed *Streptococcus pneumoniae*-related (SP-related) LRI burden, focusing on mortality, disability-adjusted life years (DALYs), and temporal trends by age, gender, geographic region, and socio-demographic index (SDI) quintiles. Decomposition analysis assessed the influence of epidemiological shifts, population growth, and aging on age-standardized mortality rates (ASMRs), while an autoregressive integrated moving average (ARIMA) model projected future trends. **Results**: Between 1990 and 2021, the global SP-related LRI death number decreased from 1,028,083 (95% uncertainty interval (UI): 923,782–1,146,074) to 505,268 (95% UI: 454,335–552,539), and the ASMR dropped from 19.28 (95% UI: 17.32–21.49) to 6.40 (95% UI: 5.76–7.00) per 100,000. The age distribution consistently exhibited a clear two-tiered pattern, gradually shifting from being predominantly composed of young children to being dominated by older adults. Disparities were stark across SDI quintiles, low-SDI regions exhibited up to 100-times-higher under-five mortality than high-SDI regions. Geographic distribution showed the highest ASMRs in sub-Saharan Africa and the lowest in Canada, the United States, and Australia, with Mongolia and Finland showing the largest reductions in mortality. Epidemiological changes were the most significant factor in ASMR reduction. **Conclusions**: The SP-related LRI burden has decreased globally but remains a major health concern, especially in low-SDI regions. Targeted public health interventions, particularly for neonates and elderly adults, are essential to address persistent disparities and further reduce mortality.

## 1. Introduction

Globally, lower respiratory infections (LRIs) are a major contributor to illness and death, disproportionately affecting vulnerable groups like young children and the elderly [1,2,3]. Among the pathogens contributing to LRI, *Streptococcus pneumoniae* (SP) stands as one of the most prevalent and deadly, causing significant mortality, particularly in resource-limited regions and among immunocompromised individuals [4]. Global initiatives, particularly the use of pneumococcal conjugate vaccines (PCVs), have been instrumental in reducing the SP-related LRI burden, especially in children under five [5,6]. These efforts have led to notable declines in LRI mortality; however, significant health disparities remain, particularly in low-socio-demographic-index (SDI) regions where vaccination coverage and healthcare access are often limited [7]. Although numerous studies address LRI trends, there is limited comprehensive analysis of SP-specific LRI burden across global, regional, and socio-economic levels. Moreover, earlier studies disproportionately concentrated on the LRI burden caused by *S. pneumoniae* (SP) in the under-five population, largely neglecting its impact in older age cohorts. Understanding these trends is critical for guiding effective interventions, particularly as demographic shifts such as aging populations and urbanization continue to impact disease patterns.

This study leverages data from the Global Burden of Disease (GBD) study to examine SP-related LRIs from 1990 to 2021. We focus on trends in mortality, DALYs, and their distribution across age groups, gender, and SDI quintiles. Additionally, we analyze the effects of epidemiological shifts, population growth, and aging factors through decomposition analysis and forecasting future trends, with the aim of identifying priority areas for intervention and reducing the health disparities associated with SP-related LRIs.

## 2. Methods

### 2.1. Data Source

Information regarding the burden of SP-related lower respiratory infections (1990–2021), such as disability-adjusted life year (DALY) numbers, mortality figures, age-standardized DALY rates, and age-standardized mortality rates, was obtained from the Global Health Data Exchange query tool (GHDx; http://ghdx.healthdata.org/gbd-results-tool; accessed on 16 August 2024). These data, disaggregated globally and by age, sex, geography, and socio-economic status, are publicly accessible via GHDx, which facilitates source identification for location-specific estimates. All analyses complied with the GBD public interest guidelines.

### 2.2. Data Stratification

The investigation assessed *S. pneumoniae*-attributable LRI burden through the stratified reporting of mortality and morbidity metrics. Absolute figures, crude incidence rates, and age-standardized rates for deaths and DALYs were generated across demographic layers: age strata (<5, 5–14, 15–49, 50–69, ≥70 years), sex, geographical location, and SDI quintiles.

DALYs serve as a comprehensive health measure, combining years of life lost (YLLs) due to early mortality with years lived with disability (YLDs), thereby encapsulating the total impact of disease [8]. The age-standardized rate is calculated based on the GBD reference population standard and is expressed as per 1000 population-years [9]. This composite metric (SDI) gauges country-level socio-economic conditions by geometrically averaging youth female fertility (<25 years), adult educational attainment (15+ years), and time-adjusted per capita income [10]. The world is categorized into five distinct regions according to their SDI levels: low (scores below 0.46), low–middle (0.46 to under 0.61), middle (0.61 to under 0.69), high–middle (0.69 to under 0.81), and high (0.81 to 1.00).

### 2.3. Autoregressive Integrated Moving Average Model

The autoregressive integrated moving average (ARIMA) model integrates concepts from both the autoregressive (AR) and moving average (MA) models. It is predicated on the assumption that a time series represents a sequence of temporally dependent random variables. ARIMA captures the autocorrelation structure within such data, enabling forecasts of future values based on historical observations. The model’s defining equation is as follows: Y_t = ϕ_1_Y_{t − 1} + ϕ_2_Y_{t − 2} + … + ϕ_pY_{t − p} + e_t − θ_1_e_{t − 1} − … − θ_qe_{t − q}. Here, the component (ϕ_1_Y_{t − 1} + … + ϕ_pY_{t − p}) constitutes the AR part (order p), while (e_t − θ_1_e_{t − 1} − … − θ_qe_{t − q}) represents the MA part (order q). Y_{t − p} denotes the observed value at time t − p, and e_t is the random error at time t. Crucially, the ARIMA requires the input time series to be transformed into a stationary, zero-mean stochastic sequence; this stationarity is typically achieved through differencing, which is the “Integrated” (I) component of the model.

### 2.4. Statistical Analysis

The annual average percent change (AAPC) was used to assess temporal trends in mortality rates from 1990 to 2021 [11]. This measure was computed for each age category, sex, and SDI quintile to detect general patterns and pinpoint notable shifts over the years. To assess the influence of demographic and epidemiological changes, a decomposition analysis of the ASMR was performed. This technique allowed us to isolate the effects of population growth, aging, and epidemiological changes on the observed burden of SP-related LRIs. An autoregressive integrated moving average (ARIMA) model was applied to the ASMR and ASDR data from 1990 to 2021 to project future trends in the SP-related LRI burden. All analyses were conducted using R software (4.2.2, R core team, R Foundation for Statistical Computing, Vienna, Austria).

## 3. Results

### 3.1. Global Trends

**Figure 1:**
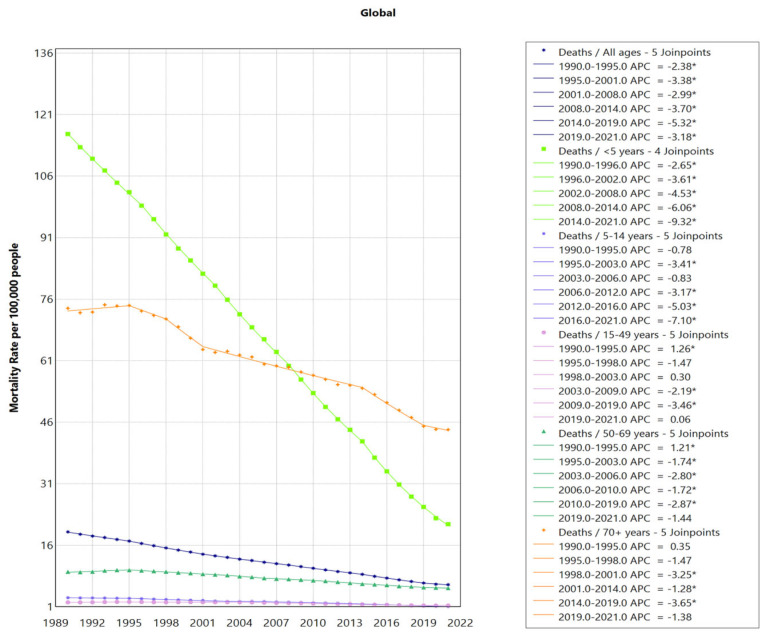
Global Trends in SP-LRI Mortality for All Ages and Selected Age Groups. LRIs = lower respiratory infections; SDI = socio-demographic index; and SP = *Streptococcus pneumoniae*. * indicates *p* < 0.05.

From 1990 to 2021, global deaths from LRIs caused by *S. pneumoniae* decreased substantially, from an estimated 1,028,083 deaths (95% uncertainty interval [UI]: 923,782–1,146,074) in 1990 to 505,268 (95% UI: 454,335–552,539) in 2021. This reduction corresponds to a decrease in the mortality rate from 19.28 per 100,000 (95% UI: 17.32–21.49) in 1990 to 6.40 per 100,000 (95% UI: 5.76–7.00) in 2021. The average annual percent change (AAPC) was −3.50% (95% CI: −3.56 to −3.43; *p* < 0.05), with the fastest decline observed from 2014 to 2019 (APC = −5.32%, *p* < 0.05) (Table 1, Table S1, Figure 1, Figure S1).

### 3.2. Trends Based on Age and Sex

Male populations generally had higher death numbers, DALY numbers, death rates, and DALY rates for SP-related LRIs across most age groups. However, among the population aged 90 and above, DALY and mortality rates were higher in females.

**Figure 2:**
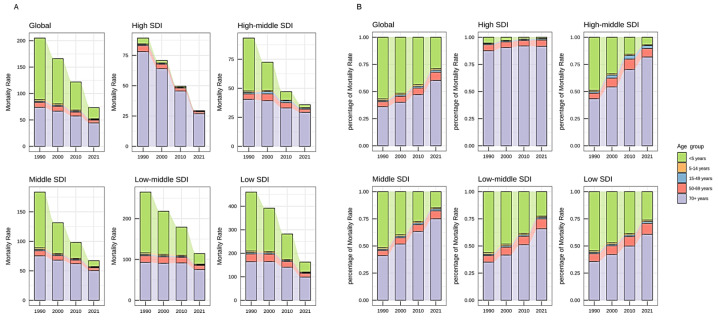
Mortality trends of SP-related LRIs, selected age groups, 1990–2021, globally and by SDI quintile. (**A**) The mortality of the selected age group; (**B**) the proportion of the selected age group’s mortality to the total population. SP = *Streptococcus pneumoniae*; LRIs = lower respiratory infections; and SDI = socio-demographic index.

By age distribution analysis, there is a decreasing trend in mortality rates across all age groups. The data revealed a clear two-tiered pattern, with younger children (under five) and elderly adults (70+ years) experiencing the highest mortality and DALY rates. However, compared to children under five years old, reducing from 116.27 (95% UI: 99.56–135.25) per 100,000 in 1990 to 21.16 (95% UI: 16.59–25.60) per 100,000 in 2021, with an AAPC of −5.40% (95% CI: −5.49 to −5.30), adults aged 70 and above experienced a slower decline in mortality rate, decreasing from 73.79 per 100,000 (95% UI: 66.94–79.70) in 1990 to 44.21 per 100,000 (95% UI: 38.92–48.22) in 2021 (AAPC = −1.62%, 95% CI: −1.97 to −1.28), making this group the one with the highest SP-related mortality in recent years (Table 1, Table S1, Figure 2).

**Figure 3:**
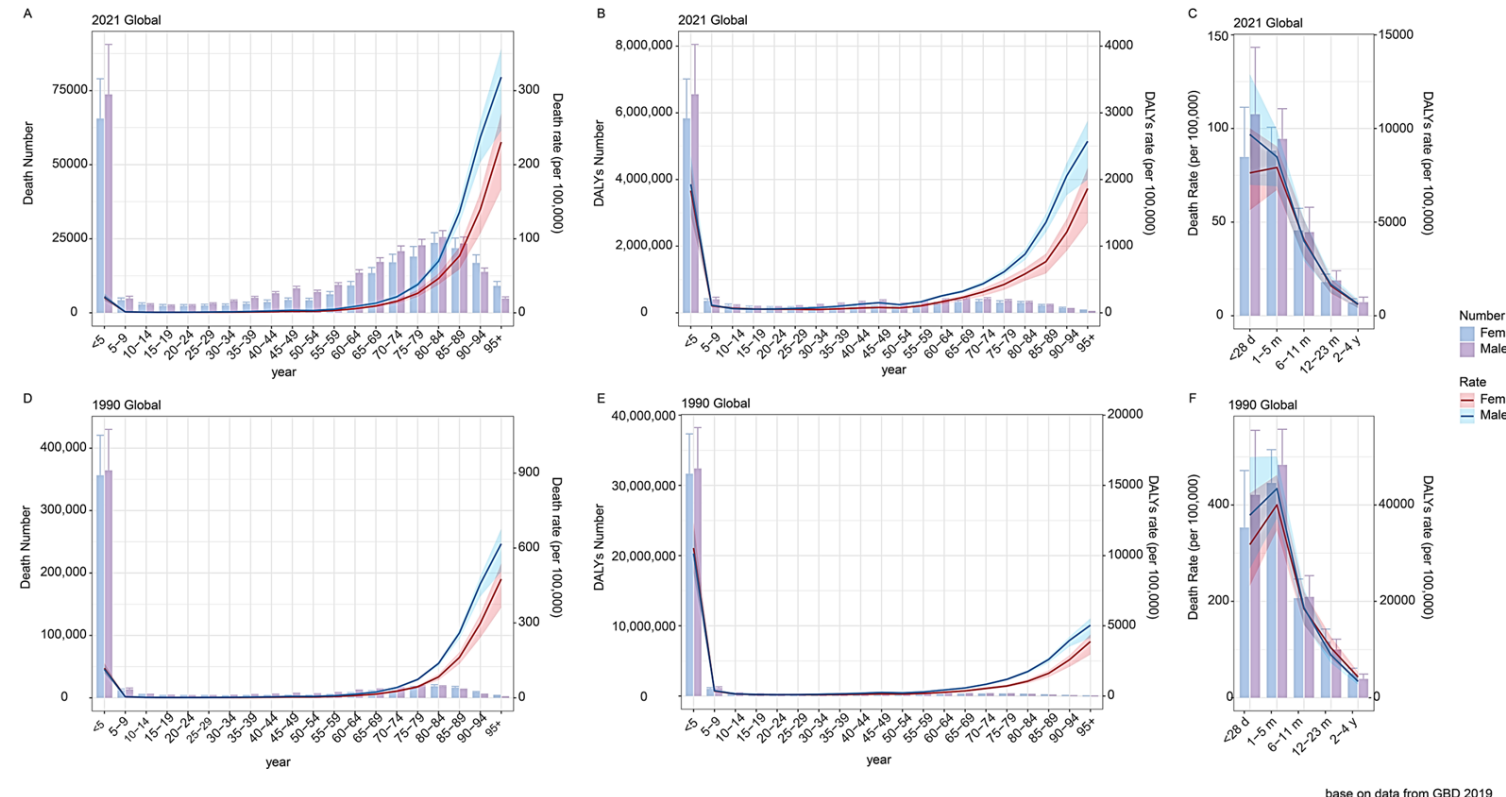
Death numbers (**A**) and DALYs (**B**) for SP-related LRI burden by age, 2021. Death numbers (**D**) and DALYs (**E**) for SP-related LRI burden by age, 1990. Death numbers for SP-related LRI burden by age under 5 years in 1990 (**C**) and 2021 (**F**). Error bars indicate the 95% uncertainty interval for numbers. Shading indicates the 95% uncertainty interval for rates. SP = *Streptococcus pneumoniae*; LRIs = lower respiratory infections; and DALYs = disability-adjusted life years.

There is a similar two-tiered differentiation in mortality and DALYs according to a narrow age group (5-year interval), but there is a clear peak in the number of deaths and DALYs in the 80–85 age group. The further stratification of children under five (<1 month, 1–5 months, 6–11 months, 12–23 months, and 2–4 years) revealed a decline in absolute neonatal mortality rates but highlighted an increasing proportional burden (Figure 3).

### 3.3. Trends Based on Socio-Demographic Index (SDI)

**Figure 4:**
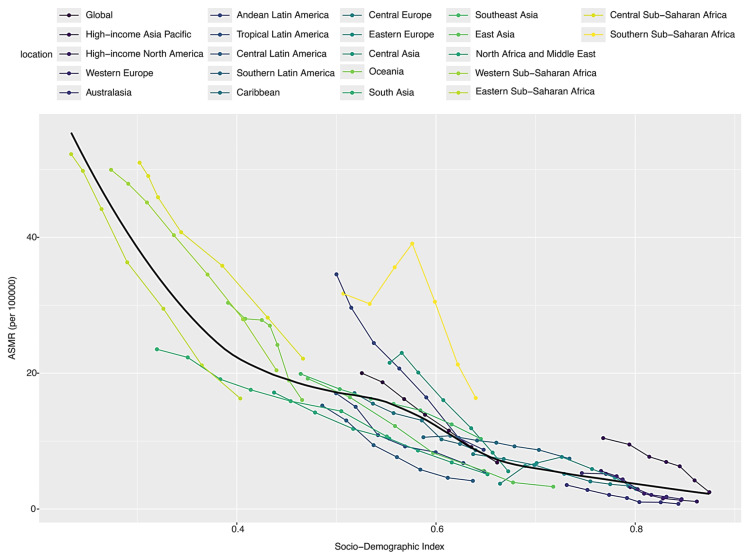
Trends in ASMR for SP-related LRI burden and SDI, globally and in 21 GBD regions. ASMRs= age-standardized mortality rates; SP = *Streptococcus pneumoniae*; LRIs = lower respiratory infections; and SDI = socio-demographic index.

Figure 4 shows that as the socio-economic index increases, the age-standardized mortality from SP-related LRIs had a decreasing trend, indicating an inverse relationship between the SDI level and ASMR. Among the 21 GBD regions, southeast sub-Saharan Africa stands out, exhibiting a significantly higher ASMR compared to the other countries and regions with a similar SDI level.

**Figure 5:**
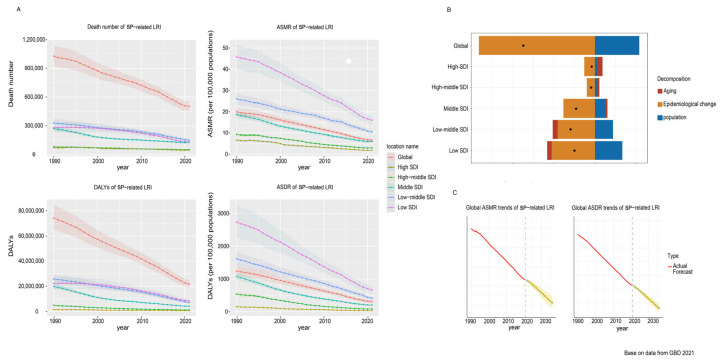
Changes in SP-related LRI ASMR according to population-level determinants of population growth, aging, and epidemiological change from 1990 to 2021 at the global level and by SDI quintile. (**A**) Numbers and age-standardized rates of deaths and DALYs for SP-related LRI burden from1990 to 2021, globally and by SDI quintile; (**B**) Changes in SP-related LRI ASMR according to population-level determinants of population growth, aging, and epidemiological change from 1990 to 2021 at the global level and by SDI quintile. The black dot represents the overall value of change contributed by all three components. For each component, the magnitude of a positive value indicates a corresponding decrease in SP-related LRI mortality attributed to the component; the magnitude of a negative value indicates a corresponding increase in SP-related LRI mortality attributed to the related component. (**C**) ASMR and ASDR trend forecast of global SP-related LRI for the next 15 years (2021–2036). The red lines represent the true values of ASMR and ASDR from 1990 to 2021, while the yellow dashed and shaded areas represent the predicted trends and 95% UI. SDI = socio-demographic index; SP = *Streptococcus pneumoniae*; LRIs = lower respiratory infections; and ASMR = age-standardized mortality rate.

Between 1990 and 2021, deaths, mortality, DALYs, and age-standardized DALY rates showed a continuous decline across SDI regions, with regional disparities gradually narrowing. The low-SDI region demonstrated the most significant and rapid decrease compared to other SDI levels, which declined most rapidly from 2014 to 2019, with an average percent change (APC) of −8.47%, but consistently recorded the highest ASMR and ASDR. For instance, in 2021, the mortality in children under five in low-SDI regions reached 42.45 per 100,000—nearly 100 times higher than that in high-SDI regions (0.34 per 100,000). In contrast, high-SDI regions maintained the lowest ASMR, with a relatively stable, gradual downward trend. In terms of death toll and DALY burden, low- and low–middle-SDI regions consistently showed higher levels than middle–high- and high-SDI regions, although the differences have gradually diminished. The middle-SDI region has maintained an intermediate level, with the rate of decline significantly slowing after 2000 (Figure 5A).

Further analysis by SDI region and age group reveals distinct trends. In medium–high-, medium–low-, and low-SDI regions, the overall trend remained consistent. Initially, individuals over 5 years old constituted the main group affected by SP-related LRIs, followed by the 70+ age group. However, the under-five population experienced the most substantial decline, ultimately making the 70+ population the primary group. In high-SDI regions, although the decline in the 70+ age group was similarly pronounced, the under- five group maintained a relatively low disease burden all the time (Figure 2, Figure S1).

### 3.4. Decomposition Analysis of Mortality Reduction Factors

From 1990 to 2021, the global number of deaths decreased by 522,815.44 cases, primarily driven by epidemiological changes, which contributed to a 161.85% reduction. Population growth partially offset these gains, contributing a 61.34% increase in deaths, while aging had a minimal impact, accounting for only a 0.51% increase. Across different SDI regions, deaths attributable to aging also varied. In high-SDI regions, aging accounted for a 163.71% relative increase in deaths; in middle-SDI regions, it accounted for an 11.54% relative increase in deaths; in low-SDI regions, aging accounted for a 22.00% relative decrease in deaths (Figure 5B). It should be noted that the GBD decomposition framework does not disaggregate the relative contributions of individual epidemiological factors (such as vaccination or healthcare access). Based on global evidence, the introduction and scale-up of PCV vaccination is likely the dominant contributor, with improvements in treatment and risk factor control also playing important roles.

An analysis across different SDI regions reveals distinct patterns. While epidemiological advancements have universally played a major role in reducing the ASMR, the impacts of aging and population growth vary significantly across SDI levels. In higher-SDI regions, aging acts as a significant barrier to further ASMR reductions. Conversely, in lower-SDI regions, aging appears to contribute positively to ASMR reduction. Meanwhile, population growth primarily hinders ASMR reduction in lower-SDI regions (Table 2).

### 3.5. Geographic Distribution of Mortality

**Figure 6:**
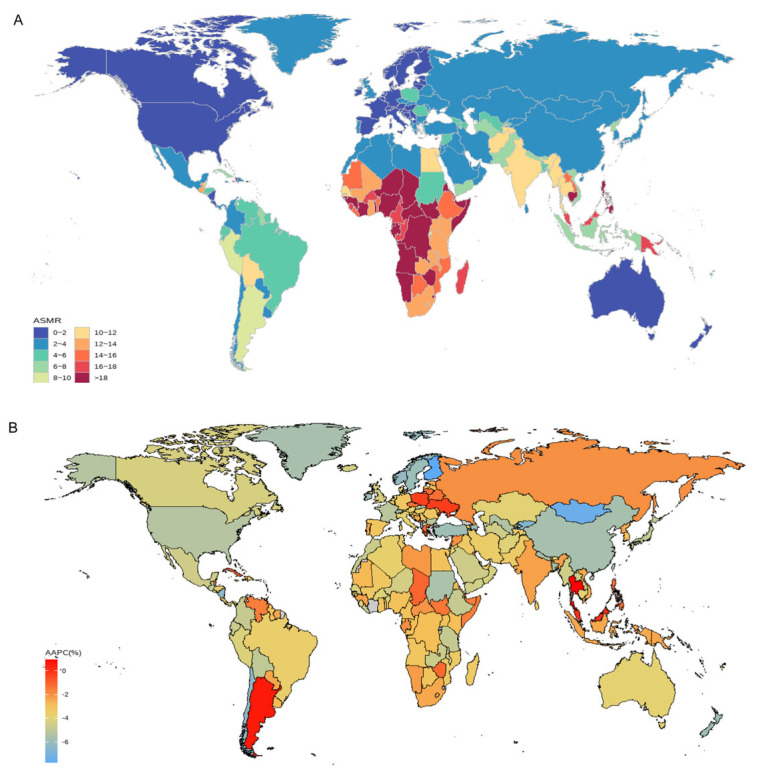
The global disease burden of SP-related LRIs for both sexes in 204 countries and territories. (**A**) The ASMR in 2021; (**B**) the AAPC of ASMR from 1990 to 2021. SP = *Streptococcus pneumoniae*; LRIs = lower respiratory infections; ASMRs = age-standardized mortality rates; and AAPC = average annual percentage change.

Using geographic mapping, we identified pronounced regional disparities in SP-related LRI mortality. The African continent exhibited the highest age-standardized mortality rates (ASMRs) for SP-related LRIs, with several sub-Saharan countries showing persistently high burdens. For several high-income countries, such as Canada, the United States, and Australia, these regions consistently had the lowest SP-related LRI mortality rates. Notable shifts in the annual average percent change (AAPC) were observed, with Mongolia and Finland showing the largest reductions in mortality between 1990 and 2021 (Figure 6).

### 3.6. Forecasted Trends

Projections using the ARIMA model indicate a continued decline in SP-related LRI ASMRs and ASDRs over the next 15 years (Figure 5C).

## 4. Discussion

This study systematically analyzed the global burden and temporal trends of lower respiratory infections (LRIs) caused by *Streptococcus pneumoniae* (SP) across age groups, genders, geographic locations, and socio-economic levels from 1990 to 2021, using data from the Global Burden of Disease (GBD) 2021 study. Our findings indicate that the global burden of LRIs due to SP has shown a steady decline from 1990 to 2021 and continues to display an “age polarization” effect, with the highest mortality rates concentrated among young children and the elderly.

The decline trend likely reflects advancements in diagnostic technologies, improvements in treatment protocols, and the impact of public health interventions, such as vaccination programs targeting pneumococcal. Generally, *S. pneumoniae* colonizes the respiratory tract asymptomatically, but weakened immunity or other health conditions can trigger the pathogen to cause severe disease [12,13]. In the early 1990s, diagnosis primarily relied on traditional culture-based methods, which often had limitations in sensitivity and took several days to yield results [14]. In the 2000s, progress in molecular diagnostics, including polymerase chain reactions (PCRs), led to quicker and more precise pathogen detection, even when the bacterial presence was minimal or cultures were challenging to cultivate [15]. In recent times, multiplex PCR and next-generation sequencing have facilitated the more extensive, high-capacity identification of pneumococcal infections, enhancing diagnostic accuracy and specificity, though they might still be out of reach in areas with limited resources [16]. The decline in mortality from SP-related LRIs is inherently the result of synergistic multifactorial effects, where advancements in diagnostic technology play a secondary role rather than serving as the primary driving force. While diagnostic technologies indirectly influence mortality by optimizing clinical decision-making, they cannot supersede the direct pathogen-clearing mechanisms of vaccines and antibiotics.

The US implemented a PCV13 vaccination strategy targeting individuals over 65, promoting it alongside influenza vaccines; research shows that PCV13 reduced all-cause pneumonia hospitalizations by 10% and lower respiratory infection hospitalizations by 9.4%, and during the COVID-19 pandemic, it lowered the risk of severe COVID-19 hospitalization and death by 30% among vaccinated seniors, highlighting its cross-protective value [17]. Japan introduced PCV7 in 2010 and switched to PCV13 within its routine program in 2013; hospital-based observational studies in Chiba Prefecture revealed that the childhood CAP incidence declined from 17.7‰ before PCV introduction in 2008 to 14.3‰ in 2012, further dropping to 10.1‰ by 2016 [18]. PCV10 was introduced in Zambia in 2013; analysis from a primary care hospital showed that total pneumonia hospitalizations in children under five fell from 108,884 cases in the 42 months before introduction to 44,715 cases in the subsequent 30 months, with PCV10 reducing pneumonia hospitalization rates by 37.8% in infants and 28.8% in children aged 1–4 [19]. Global collaboration advances vaccine development through genetic surveillance; the Gates Foundation-supported Pneumococcal Global Genome Project sequenced over 20,000 strains from 51 countries, mapping 621 genetic lineages to reveal serotype replacement patterns, informing serotype selection for next-generation vaccines to specifically target resistant and regionally prevalent strains [17].

These cases underscore that high-coverage national immunization programs, precision protection for high-risk groups, localized serotype matching, and global genetic data-driven vaccine iteration collectively form the pillars for reducing the LRI burden. The management of pneumococcal pneumonia has also undergone substantial improvements, particularly with the introduction of advanced antibiotic therapies and updated treatment guidelines. In the 1990s, treatment options were relatively limited, with penicillin and macrolide antibiotics as first-line therapies [20]. However, increasing rates of antibiotic resistance prompted research into new antibiotic classes and more nuanced treatment guidelines. The introduction of respiratory fluoroquinolones and combination therapies has enhanced treatment efficacy, especially for patients with penicillin-resistant strains [21]. In addition, recent guidelines recommend a more individualized approach to antibiotic therapy, taking into account local resistance patterns, patient comorbidities, and the severity of illness [22].

Despite these advances, several challenges remain. The SP-LRI burden remains substantial among older adults, especially those aged 70 and above, being the primary group, with a notable peak in death number for individuals aged 80–84. This finding aligns with other research suggesting that age-related immune system decline and comorbidities heighten vulnerability to respiratory infections [23]. Despite existing pneumococcal vaccination programs in high-SDI countries, significant peaks in pneumonia mortality persist among the elderly, a paradox stemming from the convergence of multiple core factors: first, a persistent immunity gap manifests as insufficient vaccine coverage and significantly weakened immune responses in the elderly—for instance, high-risk adult vaccination rates are just 4.5% in France, far below the 22.2% in the US and 17.1% in Germany [24]; second, increased disease complexity burdens the system through high comorbidity rates, frequent polymicrobial infections, and spreading antibiotic resistance, where 12.1% of elderly pneumonia cases involve polymicrobial infections, complicating diagnosis and treatment [25]. Clinical management also faces limitations, as elderly pneumonia patients often have multiple underlying conditions and face high risks of multi-organ failure; addressing this challenge requires coordinated efforts: implementing precision vaccination to boost protection, establishing integrated multidisciplinary care models for complex cases, and accelerating vaccine technology innovation to expand serotype coverage. Pneumococcal prevention strategies are profoundly reshaping global health policies for the elderly, with their impact extending across several key areas; the US Advisory Committee on Immunization Practices recommends PCV20/PCV21 immunization for all individuals aged ≥65 and those aged 19–64 with chronic conditions [26]. From a clinical practice perspective, we recommend prioritizing vaccination with the newer pneumococcal conjugate vaccine PCV20 for elderly and high-risk populations in Europe. Primary care prevention capabilities are being strengthened, as demonstrated by Austria’s integration of community clinics through an e-vaccination record system to enhance service accessibility. WHO reports indicate that increasing PCV vaccination coverage among the elderly significantly reduces antibiotic use, projecting annual savings of USD 507 million in hospitalization costs and USD 879 million in productivity losses at a 90% coverage, and novel antimicrobials like lefamulin further address treatment needs for resistant infections. Additionally, accessibility in low- and middle-income regions is being improved through price negotiations and technology transfer [27].

A particularly concerning finding is the relative increase in neonatal mortality within the under-five age group despite overall reductions in child mortality [28]. Neonates are uniquely vulnerable to severe infections due to underdeveloped immune systems, and our results suggest that existing interventions may inadequately address this critical age bracket, particularly in low-SDI regions where access to neonatal care is limited. To effectively address this persistent challenge and accelerate equitable health outcomes, we strongly recommend these targeted measures: prioritize accelerating and equalizing PCV coverage in low-SDI regions; substantially invest in strengthening primary healthcare systems, particularly empowering community health workers for the community-based recognition, management, and referral of childhood pneumonia; integrate nutritional support such as breastfeeding promotion and vitamin A/zinc supplementation with household air pollution interventions; and sustain the surveillance of pneumococcal serotype and antimicrobial resistance patterns. Additionally, perinatal care, exclusive breastfeeding support, and early neonatal infection screening should be intensified, specifically for newborns and infants. Multisectoral collaboration addressing fundamental social determinants like poverty is essential for achieving sustained and equitable reductions in SP-LRI burden. WHO and national health ministries should adopt “under-five SP-LRI mortality” and “regional ASMR” as core health resource allocation indicators while scaling up portable pulse oximeters with tiered oxygen therapy guidelines and deploying rapid pneumonia diagnostic kits in low-SDI settings to reduce antibiotic misuse.

Based on our analysis, we identified a clear inverse relationship between SDI levels and SP-LRI mortality: regions with lower SDI levels, such as sub-Saharan countries, exhibit the highest age-standardized mortality rates (ASMRs) from SP-related LRIs, while higher-SDI regions maintain the lowest ASMRs. High-income countries benefit from advanced healthcare systems, widespread vaccine availability, and higher standards of living, all of which contribute to lower mortality rates [29]. These settings allow for timely medical interventions and effective antibiotic treatments that mitigate severe outcomes and reduce mortality risk. Additionally, robust public health infrastructure in high-income countries helps prevent SP transmission and protects at-risk populations. Conversely, low-income regions encounter numerous obstacles in managing SP-related LRIs. Insufficient medical resources, under-resourced healthcare systems, low vaccination rates, and a lack of effective antibiotics all contribute to the elevated LRI mortality rates in these settings. Factors such as malnutrition, crowded living conditions, and inadequate sanitation further exacerbate the severity and transmission of pneumococcal infections [30]. Addressing these issues requires global support, including increased funding, vaccine donations, and infrastructure development in healthcare and public health systems. Strengthening preventive measures in low-income settings is essential for narrowing the health disparity gap and improving survival outcomes [31].

The GBD database remains an invaluable tool for public health policymaking, as it assesses the prevalence and impact of diseases, injuries, and risk factors worldwide. However, it is important to acknowledge some of the limitations of the GBD data. The accuracy of GBD estimates is dependent on the quality of data available from individual countries, which can vary widely. In low-SDI countries with limited resources, health data may be scarce or inconsistent, potentially resulting in underestimates of disease burden. Furthermore, data collection and reporting standards vary between countries, affecting data comparability. The GBD database provides data at the national level, which may obscure significant health disparities at subnational or community levels. This limitation underscores the need for more granular data collection to support targeted public health strategies and resource allocation.

Future studies should explore specific drivers of neonatal mortality and evaluate age-specific vaccine efficacy to ensure that neonatal and elderly populations are adequately protected. Investigations into regional barriers to healthcare access, particularly in sub-Saharan Africa and other low-SDI regions, could inform more effective intervention strategies.

## 5. Conclusions

This study demonstrates a notable global reduction in the SP-related LRI burden over the past 30 years, yet significant disparities persist, particularly in low-SDI regions and among vulnerable populations. Neonates and the elderly remain at highest risk, emphasizing the need for targeted public health interventions. Achieving further reductions will require sustained commitment to improving healthcare access, expanding vaccination coverage, and addressing socio-economic barriers. The findings provide actionable insights for policymakers to design evidence-based interventions aimed at mitigating the burden of SP-related LRIs globally, with a special focus on addressing inequities in low-resource settings.

## Figures and Tables

**Table 1:** SP-related LRI death counts and mortality rates for all ages and selected age groups in 1990 and 2021, globally and by SDI quintile, and its temporal trends from 1990 to 2021. SP = *Streptococcus pneumoniae*; LRIs = lower respiratory infections; SDI = socio-demographic index; and AAPC= annual average percent change.

Measure	1990		2021		1990–2021 AAPC, *n* (95% UI)
	Death Count, *n* (95% UI)	Mortality Rate per 100,000 People, *n* (95% UI)	Death Count, *n* (95% UI)	Mortality Rate per 100,000 People, *n* (95% UI)	
Global					
All ages	1,028,083 (923,782–1,146,074)	19.28 (17.32–21.49)	505,268 (454,335–552,539)	6.40 (5.76–7.00)	−3.50 (−3.56–−3.43)
<5 years	720,784 (617,218–838,466)	116.27 (99.56–135.25)	139,267 (109,208–168,510)	21.16 (16.59–25.60)	−5.40 (−5.49–−5.30)
5–14 years	36,176 (30,292–40,642)	3.23 (2.71–3.63)	14,651 (12,468–16,556)	1.08 (0.92–1.22)	−3.52 (−3.89–−3.14)
15–49 years	56,991 (52,591–60,815)	2.10 (1.94–2.24)	52,629 (48,293–58,005)	1.33 (1.22–1.47)	−1.44 (−1.73–−1.15)
50–69 years	65,075 (60,073–69,845)	9.54 (8.81–10.24)	80,180 (72,586–86,471)	5.58 (5.05–6.02)	–1.68 (–1.93–−1.43)
70+ years	149,057 (135,227–160,992)	73.79 (66.94–79.70)	218,540 (192,403–238,383)	44.21 (38.92–48.22)	−1.62 (–1.97–−1.28)
China					
All ages	165,798 (143,691–189,099)	14.09 (12.21–16.07)	51,180 (42,055–62,158)	3.60 (2.96–4.37)	−4.39 (–4.70–−4.08)
<5 years	118,818 (100,464–140,162)	106.27 (89.86–125.36)	3406 (2674–4283)	4.39 (3.44–5.51)	−9.80 (–10.44–−9.15)
5–14 years	5191 (4118–5917)	2.51 (1.99–2.86)	574 (484–692)	0.32 (0.27–0.38)	−6.63 (–7.07–−6.19)
15–49 years	8318 (6790–9523)	1.25 (1.02–1.43)	2444 (1983–3025)	0.37 (0.30–0.46)	−3.93 (–4.35–−3.50)
50–69 years	9202 (7595–10,630)	5.99 (4.94–6.92)	5676 (4482–7118)	1.49 (1.18–1.87)	–4.45 (−4.75–−4.15)
70+ years	24,269 (19,549–27,249)	64.67 (52.09–72.61)	39,080 (31,162–48,040)	32.76 (26.12–40.27)	−2.25 (−2.68–−1.82)
High SDI					
All ages	69,715 (63,660–73,102)	7.93 (7.24–8.31)	45,236 (38,317–49,203)	4.13 (3.50–4.50)	−2.04 (−2.67–−1.40)
<5 years	2944 (2671–3328)	4.77 (4.33–5.39)	184 (163–203)	0.34 (0.30–0.38)	−8.14 (−8.42–−7.86)
5–14 years	536 (501–575)	0.43 (0.40–0.46)	82 (77–89)	0.07 (0.06–0.07)	–5.78 (–6.52––5.04)
15–49 years	4047 (3940–4160)	0.88 (0.86–0.90)	1662 (1558–1788)	0.33 (0.31–0.36)	−3.19 (−3.76–−2.61)
50–69 years	8247 (7977–8452)	5.03 (4.87–5.16)	4725 (4513–4901)	1.71 (1.64–1.78)	−3.41 (−3.88–−2.93)
70+ years	53,941 (48,126–57,144)	78.11 (69.69–82.75)	38,584 (31,866–42,384)	26.89 (22.21–29.54)	−3.52 (–4.10–−2.95)
High–middle SDI					
All ages	80,557 (74,119–88,368)	7.57 (6.97–8.31)	51,642 (46,242–57,229)	3.96 (3.55–4.39)	−2.14 (–2.61–−1.67)
<5 years	42,282 (37,158–49,371)	45.51 (40.00–53.14)	1738 (1442–2086)	2.48 (2.06–2.98)	−9.01 (–9.31–−8.70)
5–14 years	2404 (2194–2628)	1.33 (1.21–1.45)	400 (365–458)	0.25 (0.23–0.28)	−5.42 (−5.94–−4.90)
15–49 years	6905 (6455–7316)	1.22 (1.14–1.30)	5893 (5489–6303)	0.94 (0.87–1.00)	−0.94 (−2.08–0.21)
50–69 years	8153 (7612–8685)	4.68 (4.37–4.99)	9240 (8572–9930)	2.83 (2.63–3.04)	−0.65 (−2.53–−0.77)
70+ years	20,813 (18,612–22,452)	40.44 (36.17–43.63)	34,372 (29,394–39,045)	29.28 (25.04–33.26)	−1.09 (−1.39–−0.79)
Middle SDI					
All ages	269,994 (246,668–294,963)	15.67 (14.32–17.12)	129,690 (117,334–140,965)	5.30 (4.79–5.76)	−3.47 (−3.60–−3.34)
<5 years	189,460 (169,317–214,221)	94.48 (84.43–106.82)	17,638 (14,720–20,934)	9.99 (8.33–11.85)	−7.11 (−7.36–−6.86)
5–14 years	10,738 (9029–11,704)	2.85 (2.40–3.11)	2690 (2442–2966)	0.69 (0.63–0.76)	−4.55 (−4.83–−4.27)
15–49 years	18,830 (17,417–19,984)	2.07 (1.91–2.19)	14,271 (13,292–15,356)	1.14 (1.06–1.22)	−1.99 (−2.42–−1.54)
50–69 years	16,513 (15,045–17,894)	8.72 (7.94–9.45)	24,093 (22,076–25,955)	4.96 (4.55–5.34)	−1.81 (–2.00–−1.62)
70+ years	34,452 (30,871–37,898)	75.40 (67.56–82.94)	70,998 (61,361–78,348)	50.36 (43.52–55.57)	−1.29 (–1.60–−0.98)
Low–middle SDI					
All ages	32,8670 (289,275–372,053)	28.30 (24.91–32.03)	149,827 (132,279–165,722)	7.80 (6.89–8.63)	−4.08 (−4.24–−3.93)
<5 years	258,180 (220,990–30,0934)	148.82 (127.38–173.47)	49,278 (40,015–59,051)	25.72 (20.89–30.82)	−5.56 (−5.75–−5.37)
5–14 years	12,833 (10,658–14,838)	4.30 (3.57–4.97)	4827 (4109–5478)	1.24 (1.06–1.41)	−4.00 (−4.63–−3.37)
15–49 years	15,135 (13,615–17,100)	2.75 (2.47–3.10)	16,198 (14,400–18,419)	1.59 (1.42–1.81)	−1.75 (−1.92–−1.57)
50–69 years	18,140 (16,119–20,134)	16.20 (14.39–17.98)	26,805 (23,433–29,939)	10.51 (9.19–11.74)	−1.33 (−1.44–−1.22)
70+ years	24,383 (21,309–28,620)	92.98 (81.26–109.13)	52,718 (46,007–59,006)	75.21 (65.64–84.18)	−0.71 (−1.14–−0.28)
Low SDI					
All ages	278,423 (228,177–335,705)	55.54 (45.52–66.97)	128,394 (105,928–150,956)	11.49 (9.48–13.51)	−4.96 (−5.06–−4.86)
<5 years	227,480 (176,711–284,306)	250.54 (194.62–313.13)	70,291 (51,377–89,236)	42.45 (31.03–53.89)	−5.60 (−5.72–−5.48)
5–14 years	9644 (7322–11,670)	6.98 (5.30–8.45)	6643 (5330–7837)	2.25 (1.81–2.66)	−3.58 (−4.11–−3.05)
15–49 years	12,019 (10,420–13,685)	5.44 (4.71–6.19)	14,557 (12,524–16,735)	2.68 (2.31–3.09)	−2.26 (−2.42–−2.10)
50–69 years	13,960 (12,046–15,873)	33.23 (28.67–37.78)	15,245 (13,226–17,316)	16.42 (14.24–18.65)	−2.24 (−2.36–−2.12)
70+ years	15,319 (13,320–17,666)	164.16 (142.73–189.31)	21,657 (19,072–25,252)	98.74 (86.95–115.13)	−1.60 (−1.78–−1.41)

**Table 2:** Changes in SP-related LRI ASMR according to population-level determinants of population growth, aging, and epidemiological change from 1990 to 2021 at the global level and by SDI quintile. SDI = socio-demographic index; SP = respiratory syncytial virus; LRIs = lower respiratory infections; and ASMRs = age-standardized mortality rates.

Location	Overall Difference ^a^	Change Due to Population-Level Determinants		
		(% Contribution to the Total Changes)		
		Aging ^b^	Population ^c^	Epidemiological Change ^d^
Global	−522,815.44	2671.32 (−0.51%)	320,706.21 (−61.34%)	−846,192.97 (161.85%)
SDI				
High SDI	−24,479.11	40,074.30 (−163.71%)	14,519.82 (−59.32%)	−79,073.23 (323.02%)
High–middle SDI	−28,914.83	17,228.25 (−59.58%)	13,702.65 (−47.39%)	−59,845.72 (206.97%)
Middle SDI	−140,304.26	16,194.96 (−11.54%)	73,470.84 (−52.37%)	−229,970.06 (163.91%)
Low–middle SDI	−178,843.08	−35,367.88 (19.78%)	129,947.81 (−72.66%)	−273,423.00 (152.88%)
Low SDI	−150,029.57	−33,001.29 (22.00%)	199,574.12 (−133.02%)	−316,602.39 (211.03%)

^a.^ Change in mortality between years 2021 and 1990. ^b^ Change in mortality due to change in the age structure. ^c^ Change in mortality due to change in population number. ^d^ Change in mortality due to epidemiologic changes. Epidemiologic changes refer to the mortality change when age structure and population hold constant.

## Data Availability

All data analyzed in this study is shared publicly on the GBD website.

## Supplementary Materials

The following supporting information can be downloaded at https://www.mdpi.com/article/10.3390/healthcare13161982/s1, Table S1: SP-related LRI death counts and mortality rates for all ages and selected age groups in 1990 and 2021, globally and by SDI quintile, and its temporal trends from 1990 to 2021. SP = *Streptococcus pneumoniae*; LRIs = lower respiratory infections; SDI = socio-demographic index; and AAPC = annual average percent change. Figure S1: Trends in global (A), high-SDI (B), high–middle-SDI (C), middle-SDI (D), low–middle-SDI (E), and low-SDI (F) regions of SP-LRI death number for all ages and selected age groups. LRIs = lower respiratory infections; SDI = socio-demographic index; and SP = *Streptococcus pneumoniae*. * indicates *p* < 0.05.

## Abbreviations

The following abbreviations are used in this manuscript:

LRIs	Lower respiratory infections
SP	*Streptococcus pneumoniae*
GBD	Global Burden of Disease
DALYs	Disability-adjusted life years
SDI	Socio-demographic index
ASMRs	Age-standardized mortality rates
AAPC	Annual average percent change
