# Supplementary material for: The Global, Regional, and National Burden of Lower Respiratory Infections Caused by Streptococcus pneumoniae Between 1990 and 2021
Source: Healthcare (Basel). 2025 Aug 12;13(16):1982. doi: 10.3390/healthcare13161982 (PMC12385783; doi:10.3390/healthcare13161982)
Supplement: Supplementary file 1 [file healthcare-13-01982-s001.zip › healthcare-3749724-supplementary.pdf]

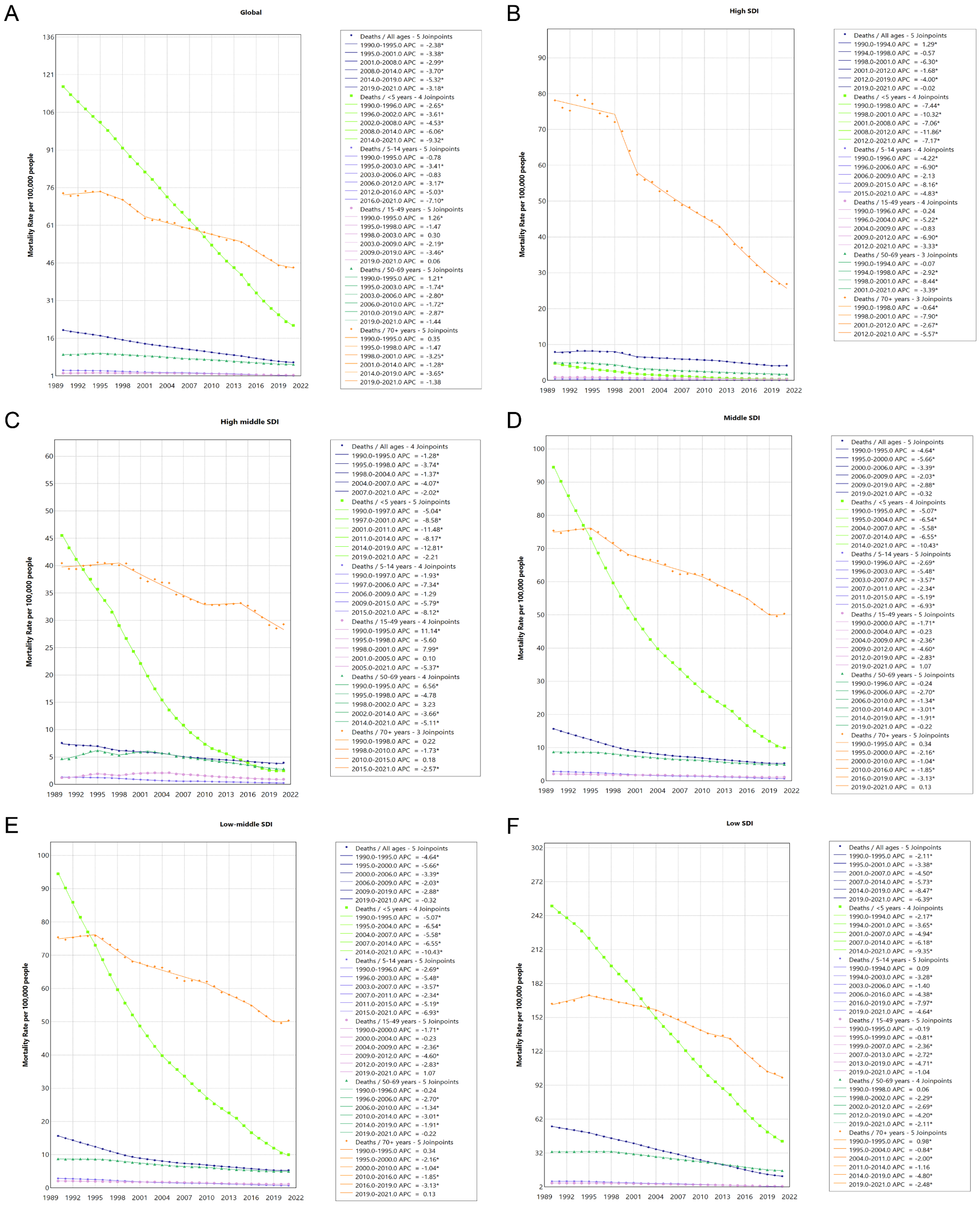

Figure S1. Trends in global (**A**), high-SDI (**B**), high–middle-SDI (**C**), middle-SDI (**D**), low–middle-SDI (**E**), and low-SDI (**F**) regions of SP-LRI death number for all ages and selected age groups. LRIs = lower respiratory infections; SDI = socio-demographic index; and SP = *Streptococcus pneumoniae*. * indicates $p < 0.05$.

Table S1. SP-related LRI death counts and mortality rates for all ages and selected age groups in 1990 and 2021, globally and by SDI quintile, and its temporal trends from 1990 to 2021. SP = *Streptococcus pneumoniae*; LRIs = lower respiratory infections; SDI = socio-demographic index; and AAPC= annual average percent change.

	1990		2000		2010		2021		
**Measure**	**Deaths count *n* (95% UI)**	**Mortality Rate per 100,000 people *n* (95% UI)**	**Deaths count *n* (95% UI)**	**Mortality Rate per 100,000 people *n* (95% UI)**	**Deaths count *n* (95% UI)**	**Mortality Rate per 100,000 people *n* (95% UI)**	**Deaths count *n* (95% UI)**	**Mortality Rate per 100,000 people *n* (95% UI)**	**1990–2021 AAPC *n* (95% UI)**
Global									
All ages	1028083 (923782-1146074)	19.28 (17.32-21.49)	875915 (798301-959610)	14.36 (13.09-15.73)	724190 (663755-788344)	10.42 (9.55-11.34)	505268 (454335-552539)	6.40 (5.76-7.00)	-3.50 (-3.56--3.43)
<5 years	720784 (617218-838466)	116.27 (99.56-135.25)	520273 (449003-597886)	85.46 (73.75-98.21)	348116 (299939-402328)	53.10 (45.76-61.37)	139267 (109208-168510)	21.16 (16.59-25.60)	-5.40 (-5.49--5.30)
5-14 years	36176 (30292-40642)	3.23 (2.71-3.63)	32286 (27778-35847)	2.63 (2.27-2.93)	24367 (21271-27418)	2.00 (1.75-2.25)	14651 (12468-16556)	1.08 (0.92-1.22)	-3.52 (-3.89--3.14)
15-49 years	56991 (52591-60815)	2.10 (1.94-2.24)	69122 (64718-73177)	2.17 (2.03-2.29)	66660 (62326-71294)	1.82 (1.70-1.95)	52629 (48293-58005)	1.33 (1.22-1.47)	-1.44 (-1.73--1.15)
50-69 years	65075 (60073-69845)	9.54 (8.81-10.24)	74121 (69043-78666)	9.21 (8.58-9.77)	78936 (73751-84104)	7.44 (6.95-7.93)	80180 (72586-86471)	5.58 (5.05-6.02)	-1.68 (-1.93--1.43)
70+ years	149057 (135227-160992)	73.79 (66.94-79.70)	180113 (161728-194650)	66.51 (59.72-71.88)	206113 (183268-223285)	57.44 (51.07-62.22)	218540 (192403-238383)	44.21 (38.92-48.22)	-1.62 (-1.97--1.28)
China									
All ages	165798 (143691-189099)	14.09 (12.21-16.07)	88448 (80297-95327)	7.02 (6.38-7.57)	49535 (44378-56953)	3.71 (3.32-4.26)	51180 (42055-62158)	3.60 (2.96-4.37)	-4.39 (-4.70--4.08)
<5 years	118818 (100464-140162)	106.27 (89.86-125.36)	40396 (35544-45406)	52.28 (46.00-58.77)	8952 (7722-10324)	11.92 (10.29-13.75)	3406 (2674-4283)	4.39 (3.44-5.51)	-9.80 (-10.44--9.15)
5-14 years	5191 (4118-5917)	2.51 (1.99-2.86)	3010 (2525-3332)	1.39 (1.17-1.54)	976 (890-1096)	0.67 (0.61-0.75)	574 (484-692)	0.32 (0.27-0.38)	-6.63 (-7.07--6.19)
15-49 years	8318 (6790-9523)	1.25 (1.02-1.43)	6402 (5610-6979)	0.88 (0.78-0.96)	3623 (3229-4245)	0.47 (0.42-0.55)	2444 (1983-3025)	0.37 (0.30-0.46)	-3.93 (-4.35--3.50)

50-69 years	9202 (7595-10630)	5.99 (4.94-6.92)	7844 (6874-8752)	4.18 (3.66-4.66)	5275 (4675-6227)	2.02 (1.79-2.38)	5676 (4482-7118)	1.49 (1.18-1.87)	-4.45 (-4.75--4.15)
70+ years	24269 (19549-27249)	64.67 (52.09-72.61)	30798 (26475-33774)	57.32 (49.27-62.86)	30709 (26642-35991)	40.36 (35.01-47.30)	39080 (31162-48040)	32.76 (26.12-40.27)	-2.25 (-2.68--1.82)
High SDI									
All ages	69715 (63660-73102)	7.93 (7.24-8.31)	67872 (60415-71853)	7.19 (6.40-7.61)	58226 (50239-62499)	5.68 (4.90-6.09)	45236 (38317-49203)	4.13 (3.50-4.50)	-2.04 (-2.67--1.40)
<5 years	2944 (2671-3328)	4.77 (4.33-5.39)	1163 (1081-1253)	2.03 (1.89-2.19)	484 (456-513)	0.84 (0.79-0.89)	184 (163-203)	0.34 (0.30-0.38)	-8.14 (-8.42--7.86)
5-14 years	536 (501-575)	0.43 (0.40-0.46)	302 (288-323)	0.24 (0.23-0.26)	156 (150-167)	0.13 (0.13-0.14)	82 (77-89)	0.07 (0.06-0.07)	-5.78 (-6.52--5.04)
15-49 years	4047 (3940-4160)	0.88 (0.86-0.90)	3348 (3258-3437)	0.69 (0.67-0.71)	2498 (2412-2579)	0.49 (0.48-0.51)	1662 (1558-1788)	0.33 (0.31-0.36)	-3.19 (-3.76--2.61)
50-69 years	8247 (7977-8452)	5.03 (4.87-5.16)	7138 (6906-7317)	3.76 (3.63-3.85)	5926 (5713-6081)	2.50 (2.41-2.57)	4725 (4513-4901)	1.71 (1.64-1.78)	-3.41 (-3.88--2.93)
70+ years	53941 (48126-57144)	78.11 (69.69-82.75)	55922 (48566-59790)	64.03 (55.61-68.46)	49161 (41345-53394)	45.61 (38.36-49.54)	38584 (31866-42384)	26.89 (22.21-29.54)	-3.52 (-4.10--2.95)
High-middle SDI									
All ages	80557 (74119-88368)	7.57 (6.97-8.31)	70372 (66780-73733)	6.10 (5.79-6.40)	57909 (54037-62185)	4.71 (4.40-5.06)	51642 (46242-57229)	3.96 (3.55-4.39)	-2.14 (-2.61--1.67)
<5 years	42282 (37158-49371)	45.51 (40.00-53.14)	17123 (15550-18958)	24.31 (22.08-26.92)	5330 (4809-5907)	7.32 (6.61-8.12)	1738 (1442-2086)	2.48 (2.06-2.98)	-9.01 (-9.31--8.70)
5-14 years	2404 (2194-2628)	1.33 (1.21-1.45)	1681 (1552-1823)	0.94 (0.87-1.02)	754 (709-832)	0.54 (0.51-0.60)	400 (365-458)	0.25 (0.23-0.28)	-5.42 (-5.94--4.90)
15-49 years	6905 (6455-7316)	1.22 (1.14-1.30)	12571 (12238-12989)	1.96 (1.91-2.03)	10711 (10469-11082)	1.57 (1.54-1.63)	5893 (5489-6303)	0.94 (0.87-1.00)	-0.94 (-2.08-0.21)
50-69 years	8153 (7612-8685)	4.68 (4.37-4.99)	11323 (10916-11833)	5.91 (5.69-6.17)	11143 (10768-11626)	4.53 (4.38-4.73)	9240 (8572-9930)	2.83 (2.63-3.04)	-1.65 (-2.53--0.77)
70+ years	20813 (18612-22452)	40.44 (36.17-43.63)	27674 (24744-30094)	39.28 (35.12-42.71)	29972 (26550-33078)	33.06 (29.29-36.49)	34372 (29394-39045)	29.28 (25.04-33.26)	-1.09 (-1.39--0.79)

Middle SDI									
All ages	269994 (246668-294963)	15.67 (14.32-17.12)	182657 (170757-193047)	9.34 (8.73-9.87)	150837 (140501-160485)	6.87 (6.40-7.31)	129690 (117334-140965)	5.30 (4.79-5.76)	-3.47 (-3.60--3.34)
<5 years	189460 (169317-214221)	94.48 (84.43-106.82)	93295 (84861-102273)	52.05 (47.34-57.06)	49151 (43300-55401)	26.90 (23.70-30.32)	17638 (14720-20934)	9.99 (8.33-11.85)	-7.11 (-7.36--6.86)
5-14 years	10738 (9029-11704)	2.85 (2.40-3.11)	7865 (6969-8454)	1.94 (1.72-2.09)	4785 (4360-5174)	1.32 (1.20-1.43)	2690 (2442-2966)	0.69 (0.63-0.76)	-4.55 (-4.83--4.27)
15-49 years	18830 (17417-19984)	2.07 (1.91-2.19)	19089 (18054-20440)	1.78 (1.69-1.91)	18327 (17285-19530)	1.49 (1.41-1.59)	14271 (13292-15356)	1.14 (1.06-1.22)	-1.99 (-2.42--1.54)
50-69 years	16513 (15045-17894)	8.72 (7.94-9.45)	18093 (16892-19474)	7.66 (7.15-8.24)	20354 (19040-21539)	6.17 (5.78-6.53)	24093 (22076-25955)	4.96 (4.55-5.34)	-1.81 (-2.00--1.62)
70+ years	34452 (30871-37898)	75.40 (67.56-82.94)	44315 (39835-48864)	68.08 (61.20-75.07)	58219 (51985-63614)	62.08 (55.43-67.83)	70998 (61361-78348)	50.36 (43.52-55.57)	-1.29 (-1.60--0.98)
Low-middle SDI									
All ages	328670 (289275-372053)	28.30 (24.91-32.03)	279933 (251957-309578)	20.13 (18.12-22.26)	235378 (213804-257898)	14.33 (13.01-15.70)	149827 (132279-165722)	7.80 (6.89-8.63)	-4.08 (-4.24--3.93)
<5 years	258180 (220990-300934)	148.82 (127.38-173.47)	194848 (170238-220731)	105.03 (91.77-118.98)	135445 (118502-154333)	68.76 (60.16-78.35)	49278 (40015-59051)	25.72 (20.89-30.82)	-5.56 (-5.75--5.37)
5-14 years	12833 (10658-14838)	4.30 (3.57-4.97)	12172 (10296-13860)	3.59 (3.04-4.09)	9082 (7875-10475)	2.48 (2.15-2.86)	4827 (4109-5478)	1.24 (1.06-1.41)	-4.00 (-4.63--3.37)
15-49 years	15135 (13615-17100)	2.75 (2.47-3.10)	18872 (17415-21114)	2.72 (2.51-3.04)	19475 (17897-21784)	2.30 (2.11-2.57)	16198 (14400-18419)	1.59 (1.42-1.81)	-1.75 (-1.92--1.57)
50-69 years	18140 (16119-20134)	16.20 (14.39-17.98)	21719 (19482-23942)	15.89 (14.25-17.52)	25490 (23224-27614)	14.00 (12.76-15.17)	26805 (23433-29939)	10.51 (9.19-11.74)	-1.33 (-1.44--1.22)
70+ years	24383 (21309-28620)	92.98 (81.26-109.13)	32321 (28548-37261)	90.63 (80.05-104.48)	45888 (40525-51449)	91.55 (80.85-102.64)	52718 (46007-59006)	75.21 (65.64-84.18)	-0.71 (-1.14--0.28)
Low SDI									
All ages	278423 (228177-335705)	55.54 (45.52-66.97)	274405 (230629-325163)	42.21 (35.48-50.02)	221264 (190920-254752)	25.96 (22.40-29.89)	128394 (105928-150956)	11.49 (9.48-13.51)	-4.96 (-5.06--4.86)

<5 years	227480 (176711-284306)	250.54 (194.62-313.13)	213505 (171448-263488)	184.14 (147.87-227.25)	157449 (131106-189764)	108.50 (90.35-130.77)	70291 (51377-89236)	42.45 (31.03-53.89)	-5.60 (-5.72--5.48)
5-14 years	9644 (7322-11670)	6.98 (5.30-8.45)	10248 (8047-12062)	5.78 (4.54-6.80)	9576 (8016-10977)	4.09 (3.43-4.69)	6643 (5330-7837)	2.25 (1.81-2.66)	-3.58 (-4.11--3.05)
15-49 years	12019 (10420-13685)	5.44 (4.71-6.19)	15181 (13209-17114)	5.14 (4.47-5.80)	15595 (13505-17523)	3.98 (3.44-4.47)	14557 (12524-16735)	2.68 (2.31-3.09)	-2.26 (-2.42--2.10)
50-69 years	13960 (12046-15873)	33.23 (28.67-37.78)	15782 (13940-17641)	31.86 (28.14-35.61)	15956 (14060-17990)	24.57 (21.65-27.70)	15245 (13226-17316)	16.42 (14.24-18.65)	-2.24 (-2.36--2.12)
70+ years	15319 (13320-17666)	164.16 (142.73-189.31)	19690 (17223-22521)	164.66 (144.04-188.34)	22688 (20017-26607)	140.67 (124.11-164.97)	21657 (19072-25252)	98.74 (86.95-115.13)	-1.60 (-1.78--1.41)
